# Fungal natural products—the mushroom perspective

**DOI:** 10.3389/fmicb.2015.00127

**Published:** 2015-02-18

**Authors:** Marc Stadler, Dirk Hoffmeister

**Affiliations:** ^1^Department of Microbial Drugs, Helmholtz Centre for Infection ResearchBraunschweig, Germany; ^2^Department of Pharmaceutical Microbiology, Hans Knöll InstituteFriedrich Schiller Universität Jena, Germany

**Keywords:** Basidiomycete, natural product, secondary metabolism, bioactivity

Among the first documented studies on the chemistry of fungal natural products were descriptions of quinoid pigments, i.e., the L-tyrosine- and L-phenylalanine-derived terphenylquinones atromentin and polyporic acid, respectively. The isolation of these compounds from mushroom fruiting bodies (basidiomes) was published around 1877 by Stahlschmidt and Thörner. Ever since, organic chemists embraced basidiomycetes as a prolific source of bioactive compounds and investigated these fungi with regard to compound isolation, structure elucidation, and synthesis (Gill and Steglich, 1987; Zhou and Liu, 2010; De Silva et al., 2013 and previous reviews referenced therein, Lorenzen and Anke, 1998; Richter et al., in press). Mushrooms seem to be particularly talented in producing unique terpenoids, and the molecular background behind the biosynthesis of some of those compounds has only recently been elucidated (Quin et al., 2014). Prominent examples of basidiomycete metabolites for lead structures in agrochemistry and drug research are, among others, the strobilurins, i.e., agriculturally used ß-methoxyacrylate fungicides from cultures of *Mycena*, *Oudemansiella*, *Strobilurus*, *Xerula* and several other basidiomycete genera (Sauter et al., 1999, Figure 1). Other examples are the pleuromutilins, the illudins, and the omphalotins (Figure 1). The pleuromutilins from cultures of species that are now placed in the genera *Clitopilus* and *Omphalina* served as scaffold for the development of the semisynthetic antibacterial antibiotic retapamulin (Kirst, 2013) which is clinically used for topical treatment of infections with *Staphylococcus aureus*. The illudins from *Lampteromyces* and *Omphalotus* species (Omphalotaceae) are sesquiterpenes featuring an unusual cyclopropane ring and are currently developed as anticancer drugs (Tanasova and Sturla, 2012). The omphalotins are cyclopeptides with pronounced nematicidal activites against root knot nematodes (Büchel et al., 1998), which are also exclusively found in the Omphalotaceae. Recently, the blazeispirols from *Agaricus subrufescens* were discovered as strong and selective agonists of the Liver X receptor (LXR alpha). Concurrently, relevant *in vivo* effects of blazeispirols in a mouse model were observed which might give rise to the development of a new anti-hypercholesterolemic agent from cultures of a medicinal mushroom (Grothe et al., 2011).

**Figure 1:**
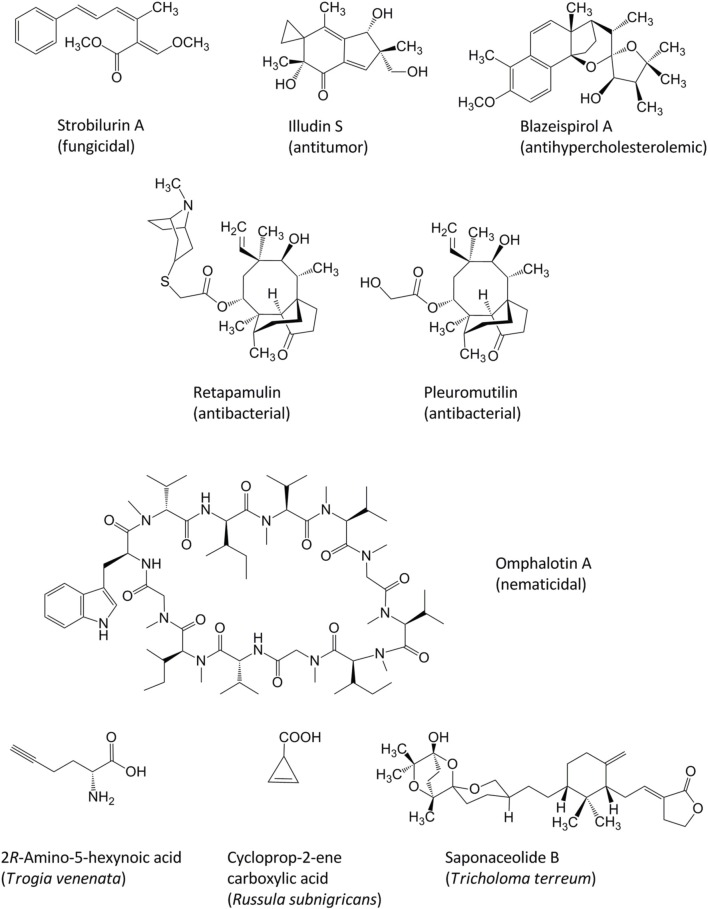
**Chemical structures of pharmacologically active basidiomycete natural products**.

The above examples illustrate that basidiomycete secondary metabolomes merit further exploration. Perhaps fortunately for coming generations of Ph.D. students, the realm of basidiomycete metabolites is still underexplored, even after decades of intensive research to isolate and structurally elucidate compounds. This is also evident by the fact that toxic principles of mushrooms which repeatedly led to poisonings were identified only recently (Figure 1). Recent advances pertain to *Trogia venenata* fruiting bodies, in which the toxic 2*R*-amino-5-hexynoic acid and related compounds were found (Zhou et al., 2012). Cycloprop-2-ene carboxylic acid causing rhabdomyolysis was isolated from *Russula subnigricans*, a toxic mushroom native to East Asia (Matsuura et al., 2009). Furthermore, saponaceolide toxins with their unusual molecular skeleton were discovered in *Tricholoma terreum* (Yin et al., 2014).

For basidiomycetes, the genomic era set in later than for ascomycetes, and in numbers of genome projects the former are still lagging behind the latter. Still, the available genomic data impacted natural product research as it reveals a stimulating disparity: the number of natural product genes, best reflected by the number of genes for polyketide synthases and peptide synthetases exceeds the number of known compounds by far—even after decades of chemical research. The “house eater” fungus *Serpula lacrymans* encodes 21 PKS and NRPS genes (Eastwood et al., 2011), the average number of PKS genes per basidiomycete genome is four, according to a survey of 35 mostly saprotrophic species (Lackner et al., 2012).

The wealth of natural product biosynthesis genes in a given species contrasts the few compounds known from the same species. This situation is reminiscent of what was found for ascomycete genomes years ago, e.g., for the genera *Aspergillus*, *Penicillium*, *Fusarium*, and others (Keller et al., 2005; Desjardins and Proctor, 2007; Sanchez et al., 2012). However, the course research has taken (and will be taking) to make as much sense as possible out of the genomic data is quite different with basidiomycetes. This is due to a number of reasons that contrast the situation with ascomycetes.

- Basidiomycetes are mostly dikaryotic and hence little suitable for reverse genetics, although some species grow as monokarya *in vitro*.
- Basidiomycetes are little amenable, if at all, to transformation and genetic manipulation, as only a very modest number of genetic tools and procedures are in place. Notable exceptions with regard to producers of pharmacologically active metabolites pertain to the honey mushroom *Armillaria mellea* (Baumgartner et al., 2010) which produces the melleolides, i.e., unusual sesquiterpene ester antibiotics (Bohnert et al., 2014). Also, a transformation procedure was established for the pleuromutilin producer *Clitopilus passeckerianus* (Kilaru et al., 2009).
- The ecological preferences of numerous basidiomycetes have so far remained obscure and can only now be established by using modern methods of molecular ecology. For instance, some species that were hitherto regarded as saprotrophs, such as *Hygrocybe virginea*, are now suspected to possess hitherto unknown associations with plants, illustrating in-depth studies on their ecology might in future be rewarding (Tello et al., 2014).
- Many basidiomycetes, in particular the biotrophic pathogens, are difficult or virtually impossible to grow in axenic culture. This applies e.g., to the entire subdivision Pucciniomycotina, (“rust fungi”) and to the obligate mycorrhizal taxa, comprising important families, such as the Russulaceae and Cortinariaceae, from whose basidiomes numerous unique secondary metabolites have already been obtained. Even in some other “saprotrophic” genera, such as *Pluteus*, the basidiospores do not readily germinate, and stable cultures can hardly be established from sterile mycelial plugs taken out of the basidome tissues using standard methodology. Protocols to culture rust fungi or mycorrhizal symbionts have been elaborated by competent mycologists several decades ago. We encourage the community to also emphasize teaching classical mycological techniques, to educate the coming generation of mycologists and prevent these valuable methods from slowly being forgotten. In fact, such techniques could be useful to facilitate work on the genomics and metabolomics of these organisms since stable cultures could be used for propagation of sufficient biomass and a number of other interesting tasks.

The typical approach to explore metabolic pathways includes gene inactivation, combined with chemical characterization of the resulting phenotype. Whereas for model species/genera such as *Aspergillus* and other ascomycetes, numerous procedures and protocols were in place for reverse genetics, to manipulate expression of silent natural product genes, and to harness -omics technologies, this is only modestly (if at all) the case for basidiomycetes. Hence, the above reasons add more complexity to research which aims at functionally characterizing individual genes and basidiomycete secondary metabolomes. Consequently, despite chemically intriguing and unique features of their natural products, and also for the lack of robust biotechnological expression systems, basidiomycetes have not become the objects of choice. As long as the respective genes, enzymes, and mechanisms are present elsewhere, e.g., in *Aspergillus* or *Fusarium* species, or in streptomycetes, these will be preferred organisms. Projects including these organisms will be sooner finished and sooner published. On the other hand, one new basidiomycete genome after the other is currently released and sequence data are made available at a much faster pace than biochemists and natural product chemists can keep up with. Hence, an increasing amount of (natural product gene) sequence data is produced and because verification by wet-bench work cannot keep pace the amount of hypothetical and misannotated natural produce genes is ever-increasing.

Despite all these challenges there are three encouraging reasons why basidiomycetes advance mycology and natural product chemistry. Firstly, unique structures, e.g., the ones mentioned in the introduction, deserve elucidation of the biochemically and mechanistically intriguing basis behind their biogenesis. Secondly, fungi as such are a widely unexplored source for novel biotechnological products in general (cf. Rambold et al., 2013), and this especially holds true for the basidiomycetes. These two reasons alone justify new genomes to be sequenced. Finally, basidiomycetes are of outstanding ecological significance and may be key to answer the question as to why natural products exist. Due to their ability to form mycorrhizae with conifers and deciduous trees, they are key elements of temperate and boreal climax vegetations. They efficiently degrade lignocellulose which makes them indispensable to keep the global carbon cycles going. The basidiomycetes and the existing genomes represent a good opportunity to follow a different approach, but still contributing substantially to natural product research: with perhaps a dozen of carefully chosen symbiotic, parasitic, and saprotrophic species and a concerted effort of mycologists, chemists, ecologists, biochemists and bioinformaticians we may come to a more profound understanding why these magnificent small molecules were evolved, beyond the established examples that they serve as defense agents and to compete with other microbes in their ecological niche.

## Conflict of interest statement

The Guest Associate Editor Nancy Keller declares that, despite having collaborated with author Dirk Hoffmeister, the review process was handled objectively. The authors declare that the research was conducted in the absence of any commercial or financial relationships that could be construed as a potential conflict of interest.
